# PUBLIC HEALTH APPROACHES FOR SUSTAINABILITY OF DEMENTIA EVIDENCE-BASED PROGRAMS

**DOI:** 10.1093/geroni/igae098.0258

**Published:** 2024-12-31

**Authors:** Kellie Waugh, Talyah Sands Leavitt

**Affiliations:** Association of State and Territorial Health Officials, Arlington, Virginia, United States; Association of State and Territorial Health Officials, Arlington, Virginia, United States

## Abstract

ASTHO, with support from CDC, has gathered strategies, examples, and resources to inform the development of sustainability focused capacity-buildings materials for public health departments, including an actionable resource for integrating sustainability into Healthy Brain Initiative (HBI) implementation. Attendees will be provided with a guided look through this tool, developed to elevate the importance of sustainability and support health departments in sustaining their programmatic dementia work. The public health-driven strategies included provide a unique guide for embedding sustainability into dementia initiatives and evidence-based programs from initial planning to implementation. Subsequently, the guidance is extended to ensure health equity is operationalized throughout the entirety of this process. Attendees will enhance their ability to ensure positive outcomes and provide long-term program benefits by thoughtfully considering complex factors that determine the sustainability of evidence-based programs, such as duration of funding, partnership, and organizational support. To encourage practical application, the presentation will showcase two state public health department examples of evidence-based programs that have effectively prioritized sustainability. The presenter will offer reflective insights on what sustainability means for public health practitioners involved with dementia and older adult health programs and shed light on how these efforts sustain positive outcomes across the life span and advance health equity.

